# A Simplified implementation of the O.U.R. stationary liquid mass balance estimation method for On-line monitoring in Animal cell production processes

**DOI:** 10.1186/1753-6561-9-S9-P56

**Published:** 2015-12-14

**Authors:** Andreu Fontova, Jonatan López-Repullo, Martí Lecina, Ramon Bragós, Jordi J Cairó

**Affiliations:** 1 Electronic Engineering Department, Universitat Politècnica de Catalunya, Barcelona, 08034, Spain; 2 Chemical Engineering Department, Universitat Autònoma de Barcelona, Cerdanyola del Vallès, 08193, Spain

## 

Many efforts have been invested in the development of culture strategies for animal cell culture process. Several strategies have been studied: batch, fed-batch and perfusion cultures [1]. For implementation of those strategies, a monitoring system for automated, controlled and optimised processes based on simple measurement could be of great interest.

Oxygen is a key substrate in animal cell metabolism and its consumption is thus a parameter of great interest for bioprocess monitoring and control. The application of the OUR (Oxygen uptake rate) is investigate here. The main advantages of OUR is that correlates well with the physiological state of cells and also for the prediction of viable cell concentration [2-4].

Different methods for the oxygen uptake rate (OUR) determination in animal cell cultivation have been developed: Dynamic estimation in the liquid phase, the global mass balance in the gas phase, and the Stationary liquid mass balance. Dynamic estimation has a considerable disadvantage because of disturbances suffered by the growing cells because of the necessary variations of the DO concentration. Gas phase balancing has several advantages; knowledge of the kL·a value is not necessary, and yields a higher density of accurate data. However, it has not historically been widely used due to the need for complex and expensive instrumentation like mass spectrometers and extremely accurate DO control systems. The Stationary liquid mass balance method offers minimum cell stress and greatest imation accuracy, but still needs for a significant investment in mass flow controllers as well as some additional instrumentation to determine the oxygen's molar fraction in the gas phase.

## Background

This works offers a simplified embodiment of the Stationary liquid mass balance method for the continuous estimate of the OUR estimation by means of the use of inexpensive proportional valves and the monitoring of their control signals is introduced and compared with the Global mass balance and Dynamic methods.

As far said electrovalves can be considered to be linear, it can be demonstrated through the Mean value theorem for integrals that the mass balance equation for the liquid phase can be expressed as a first order differential equation:

(i) $$\frac{\text{d}{\text{C}}_{\text{L}}}{\text{dt}}={\text{k}}_{\text{L}}\cdot \text{a}\cdot \left(\alpha \cdot {\text{C}}_{\text{L}}^{\text{*}}-{\text{C}}_{\text{L}}\right)-\text{OUR}$$

Where:

C_L _[mol/l]: DO concentration in the liquid phase

C*_L _[mol/l]: DO concentration in equilibrium with the gas phase

k_L_·a [1/h]: Mass transfer coefficient

a [ ]: PWM control signal or duty cycle

Now, considering the DO concentration control loop error signal for a given constant set point. The previous equation (i) can be rewritten as a function of the control loop parameters and a new expression of the OUR proportional to the duty cycle and the mass transfer coefficient can be proposed:

(ii) $${\left(\frac{\text{de}}{\text{dt}}\text{ = }\frac{{\text{dC}}_{\text{L}}^{\text{sp}}}{\text{dt}}\text{ - }\frac{\text{dCL}}{\text{dt}}\right|}_{{\text{C}}_{\text{L}}^{\text{sp}}\text{ = ctn}}\Rightarrow \frac{\text{de}}{\text{dt}}\text{ = }\frac{\text{dCL}}{\text{dt}}$$

Where:

e[mol/l]: DO control loop error signal.

C^sp^_L_[mol/l]: DO set point

(iii) $$\text{OUR}={\text{k}}_{\text{L}}\cdot \text{a}\cdot \left(\alpha \cdot {\text{C}}_{\text{L}}^{\text{*}}-{\text{C}}_{\text{L}}\left(\text{t}\right)\right)-\frac{\text{de}}{\text{dt}}$$

## Materials and methods

Three independent experiments using HEK293 cells were carried out using a 2 litre Biostat B-plus bench-scale bioreactor. The global mass balance method was applied by using a Bluesens analyzer connected to the bioreactor's gas inlet and outlet. For the Dynamic method only one external switching valve was required.

**Figure 1:**
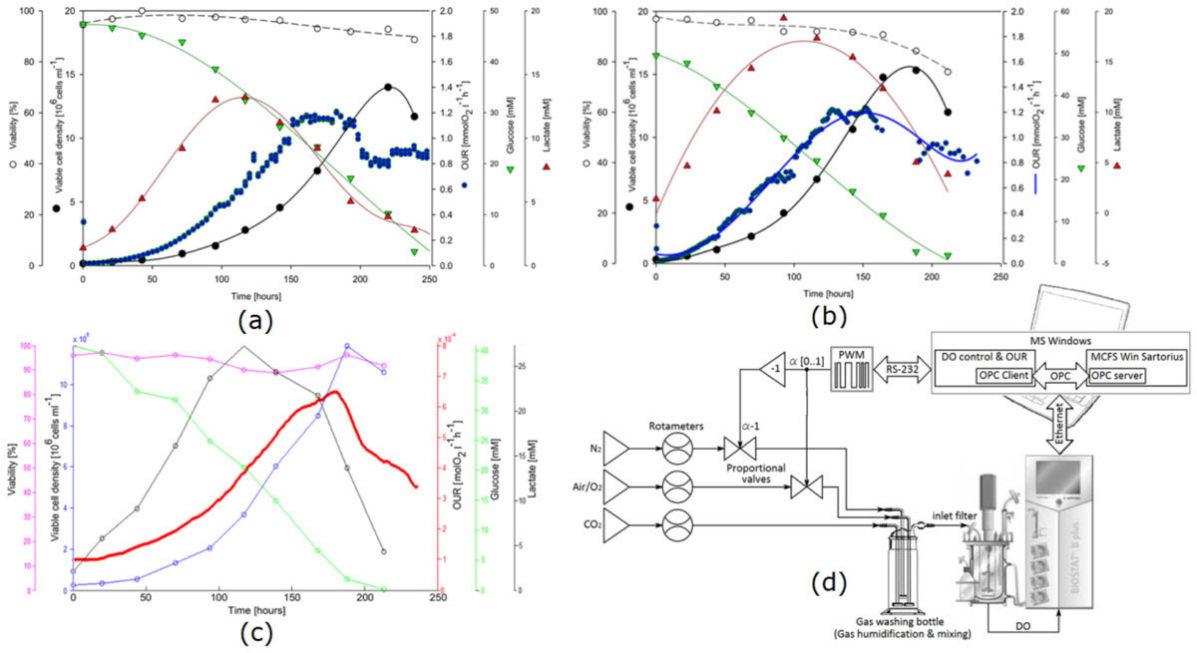
Comparison of the performance for the different OUR estimation methods (a) Dynamic method, (b) Global mass balance, (c) Simplified stationary liquid mass balance, (d) Block diagram

The simplified implementation of the liquid stationary mass balance method consisted on bypassing the Biostat B-plus system native DO control loop based on ON-OFF valves, in order to use two PWM (Pulse Width Modulation) controlled proportional valves to regulate the inlet flow of the supply gasses (Air/O2, N2), Figure 1-D.

The DO Control algorithm was executed by a customized LabWiew program where data exchange was implemented by means of an OPC server running under MFCSwin from Sartorius. Finally, the PWM drivers were also developed.

## Results

The results obtained for every experiment/estimation method displayed analogous OUR graphs.

It can be observed how for every experiment, during the first 6 days the cell concentration grown exponentially consuming glucose and producing lactate. When the remaining glucose was not enough to feed the current cell concentration a secondary metabolic path was activated and the lactate previously produced started being consumed by the cells. Once lactate became too low the oxygen consumption started to fall dawn dramatically and the number of viable cells decreased.

The off-line data available permitted a crucial observation: the fact that the turning point in the three OUR graphs allowed anticipating the time of maximum viable cell concentration (≈75 hours before). This is a very important advantage that leads to multiple possibilities regarding the culture strategy.

## Conclusions

The feasibility of the proposed OUR estimation method was demonstrated through the correlation between its performance and the results obtained for the Global mass balance and the Dynamic method for a HEK293 cell line in a bench-scale bioreactor.

In comparison with the Dynamic method the Simplified implementation of the stationary liquid mass balance shows obvious advantages in terms of time resolution and DO stability (lack of cell stress).

Regarding the method's accuracy, is expected to be comparable to the non-simplified stationary liquid mass balance due to the fact that both procedures are dependent on the previous knowledge of the mass transfer coefficient and the DO control's stationary error [5].

The results obtained can be applied to optimization of culture strategies such as fed-batch or continuous cultivation in which the proposed method can be applied.
